# Key Source Habitats and Potential Dispersal of *Triatoma infestans* Populations in Northwestern Argentina: Implications for Vector Control

**DOI:** 10.1371/journal.pntd.0003238

**Published:** 2014-10-09

**Authors:** Ricardo E. Gürtler, María C. Cecere, María del Pilar Fernández, Gonzalo M. Vazquez-Prokopec, Leonardo A. Ceballos, Juan M. Gurevitz, Uriel Kitron, Joel E. Cohen

**Affiliations:** 1 Laboratory of Eco-Epidemiology, Department of Ecology, Genetics and Evolution, Universidad de Buenos Aires-IEGEBA (CONICET-UBA), Buenos Aires, Argentina; 2 Department of Environmental Studies, Emory University, Atlanta, Georgia, United States of America; 3 Laboratory of Populations, Rockefeller and Columbia Universities, New York, New York, United States of America; Universidad Autónoma de Yucatán, Mexico

## Abstract

**Background:**

*Triatoma infestans* —the principal vector of the infection that causes Chagas disease— defies elimination efforts in the Gran Chaco region. This study identifies the types of human-made or -used structures that are key sources of these bugs in the initial stages of house reinfestation after an insecticide spraying campaign.

**Methodology and Principal Findings:**

We measured demographic and blood-feeding parameters at two geographic scales in 11 rural communities in Figueroa, northwest Argentina. Of 1,297 sites searched in spring, 279 (21.5%) were infested. Bug abundance per site and female fecundity differed significantly among habitat types (ecotopes) and were highly aggregated. Domiciles (human sleeping quarters) had maximum infestation prevalence (38.7%), human-feeding bugs and total egg production, with submaximal values for other demographic and blood-feeding attributes. Taken collectively peridomestic sites were three times more often infested than domiciles. Chicken coops had greater bug abundance, blood-feeding rates, engorgement status, and female fecundity than pig and goat corrals. The host-feeding patterns were spatially structured yet there was strong evidence of active dispersal of late-stage bugs between ecotopes. Two flight indices predicted that female fliers were more likely to originate from kitchens and domiciles, rejecting our initial hypothesis that goat and pig corrals would dominate.

**Conclusions and Significance:**

Chicken coops and domiciles were key source habitats fueling rapid house reinfestation. Focusing control efforts on ecotopes with human-fed bugs (domiciles, storerooms, goat corrals) would neither eliminate the substantial contributions to bug population growth from kitchens, chicken coops, and pig corrals nor stop dispersal of adult female bugs from kitchens. Rather, comprehensive control of the linked network of ecotopes is required to prevent feeding on humans, bug population growth, and bug dispersal simultaneously. Our study illustrates a demographic approach that may be applied to other regions and triatomine species for the design of innovative, improved vector control strategies.

## Author Summary

The major vectors of Chagas disease are species of triatomine bugs adapted to human sleeping quarters and peridomestic annexes where they feed on humans and domestic or synanthropic mammals or birds. Knowledge of the demography and nutritional status of Triatominae in real-life settings is still fragmentary, and this affects our ability to prevent or reduce house reinfestation after insecticide spraying. In addition to showing where the bugs are likely to live (occupancy and density information), our observations and analysis of flight dispersal provide insights into where bugs are likely to originate. Data on nymphal and adult sex ratios, nutritional status, and female fecundity point to the key ecotopes and sites driving the population growth of the bugs and fueling house reinfestation. Focusing control efforts on the three ecotopes (human sleeping quarters, storerooms, and goat corrals) that housed reactive, human-fed bugs would neither eliminate the substantial contributions to bug population growth from kitchens, chicken coops, and pig corrals nor stop dispersal of adult female bugs from kitchens. Rather, comprehensive control of the linked network of ecotopes in a typical house compound and community is required to prevent feeding on humans, bug population growth, and bug dispersal simultaneously.

## Introduction

Of the approximately 140 species of Triatominae (Heteroptera: Reduviidae) currently recognized, *Triatoma infestans* (Klug) expresses the extreme of an evolutionary trend toward domesticity [1], [2]. This adaptation and epidemiological significance as the most important vector of human Chagas disease justified targeting *T. infestans* for elimination in the southern cone countries of South America since 1991 [3]. Insecticide control campaigns reduced infestations but did not interrupt transmission of human *Trypanosoma cruzi* infection in the Gran Chaco region of Argentina, Bolivia and Paraguay for various reasons [4], [5].

Inadequate housing and subsistence rural economies facilitate the persistence of *T. infestans* in the Gran Chaco. Rural house compounds there typically include a dwelling house for people and all its associated special-purpose outbuildings [6]. Henceforth ‘(peri)domestic ecotopes’ will refer to human-made or -used ecotopes, either peridomestic or domestic (domestic ecotopes are sometimes called human sleeping quarters or domiciles). (Peri)domestic ecotopes are heterogeneous physically (size, materials) and demographically, with substantial variations in refuge availability, microclimatic conditions, host species composition, host density, and bug abundance per site [6]–[12].

The concept of relative habitat suitability is an important research topic that has been neglected in Triatominae. The ranked metrics of infestation prevalence and bug abundance suggested that chicken coops and goat and pig corrals were the most important ecotopes in the dry Chaco [8], [10], [11], [13]–[15], but most of these surveys did not include domiciles and the one that did include them had a different goal [13]. The current study aims to fill this knowledge gap and identify the types of (peri)domestic structures that function as key sources of bugs at the initial stages of house reinfestation after an insecticide spraying campaign.

The nutritional status of triatomine bugs (indexed by body weight-to-body length ratios, W∶L) affects all vital rates, the bugs' propensity to fly substantial distances, and the regulation of bug population size [16], [17]. The very few estimates of the blood-feeding frequency of domestic [16], [18]–[20] and peridomestic [8], [10] populations of Triatominae were restricted to *T. infestans* and *Rhodnius prolixus* Ståhl. Similarly, the only two studies that assessed variations in the nutritional status or body weight of domestic bug populations included only 3 selected houses infested by *T. infestans* and 1 house highly infested by *R. prolixus* [16], [18]. *T. infestans* from chicken coops were in better nutritional status than bug populations from pig and goat corrals [8], [10], [21], and no comparisons with domestic bug populations were ever made. With one exception [22], there is a striking lack of published information on the distributions of W∶L and female fecundity of other species of Triatominae collected in human dwellings. Estimates of female fecundity and body weight of bug populations developing in closed, small experimental huts housing 1–4 chickens [e.g.], [ 23]–[25] are less informative because variations in conditions and resources differ substantially from those in field bug populations. Whether the combined effects of habitat type and host species composition impinge on the blood-feeding rates, nutritional status and fecundity of (peri)domestic bug populations has not been investigated simultaneously in a well-defined area and is another objective of the current study.

The probability that *T. infestans* bugs initiate flight depends at least on temperature, W∶L, and season [17], [26]–[28]. Previous studies based on bug nutritional status and light-trap catches of *T. infestans* versus analysis of spatio-temporal patterns of reinfestation suggested conflicting results regarding the duration and detailed time structure of the dispersal season and subsequent establishment [10], [27], [29].

The heterogeneity of rural house compounds in the dry Chaco of Santiago del Estero Province in northern Argentina offers unique opportunities to investigate how habitat and host species may affect various population attributes of *T. infestans*. A separate article focused on the human-feeding rates of domestic *T. infestans* in the study area [20]. Here we adopted a demographic approach and measured several fitness components as putative indices of relative habitat suitability for (peri)domestic bug populations in early spring. We considered each site of occurrence of *T. infestans* as the unit of analysis to model variations in response variables. At a geographic scale including 270 houses, we measured infestation and bug abundance per site, stage structure, and host abundance. At a detailed scale including 64 infested sites, we also assessed daily blood-feeding rates, host choices, engorgement and nutritional status, and female fecundity (defined as numbers of chorionated eggs per female, including females with no chorionated eggs). We also investigated whether early spring (September–October) may represent a pulsed dispersal period of *T. infestans* [29] that previous light-trapping experiments did not detect [27].

Partial background evidence suggested that *T. infestans* would be most abundant and productive in chicken coops and other ecotopes associated with chickens (storerooms and open sheds); chicken-associated bugs would feed more often and reach a higher engorgement status, W∶L, and female fecundity than bugs from other habitats, and out-migrate much less frequently than those in pig and goat corrals [8], [10], [21], [27]. Although this ranking served as the initial hypothesis of our study, how domestic bugs and other peridomestic populations fare is uncertain given the lack of prior information. We also hypothesized that the host-feeding choices of (peri)domestic bugs should be structured by type of habitat. Testing these hypotheses is important because the current understanding of the population dynamics of Triatominae at meaningful spatial scales is still fragmentary and limits the development of improved vector control tactics.

We summarize quantitatively how the seven main ecotopes herein identified (domicile, storeroom, kitchen, chicken coop, pig corral, goat corral, granary) contribute to quantities that people may wish to control in the interest of public health: (a) the number of bugs that feed on humans (as a surrogate for the risk of human infection in different ecotopes); (b) the egg production of female bugs (as a surrogate for the contribution of different ecotopes to bug population growth); and (c) the number of dispersing females (as a surrogate for the contribution of different ecotopes to bug dispersal and reinfestation of uninfested sites).

## Materials and Methods

### Study area

Field work was carried out in the austral spring months of October–November 2003 in 11 neighboring rural communities with 270 houses in Figueroa Department (27° 23′S, 63° 29′W), Santiago del Estero Province, Argentina (Figure S1). The study area had been sprayed with pyrethroid insecticides by vector control personnel approximately three years before our fieldwork, and no further interventions were made [14]. Most houses were made of adobe walls and thatched roofs, with one or two adjacent bedrooms and a front veranda 5–10 m wide (i.e., domestic areas), and had multiple peridomestic structures as described (Text S1). Ecotope is a type of bug habitat with similar physical characteristics, function and resident hosts (e.g., chicken coop). For any house compound, a given ecotope may have more than one bug collection site (e.g., two separate chicken coops at the same house).

### Study design

A cross-sectional survey of house infestation was conducted in October–November 2003 before new control interventions [14]. Each house was visited and georeferenced, and the location and type of building material of each (peri)domestic structure were recorded.

### Vector surveys

Four teams, each composed of one supervisor and three skilled bug collectors, searched for triatomine bugs in all (peri)domestic sites of 233 inhabited houses using timed manual collections with a dislodging spray (0.2% tetramethrin, Espacial 0.2, Argentina). Two persons searched for bugs in the peridomestic ecotopes usually found infested by using 0.25 person-h on each site. Another person searched in human sleeping quarters during 30 min (0.5 person-h per domicile). On average, one person-h was used for each house compound. The ecotope where each bug was collected was classified as one of 16 types based on its function and main local host (see Results). Searches were usually conducted between 0800 and 1400 hours on non-rainy days, but sometimes searches started later because householders were not available. Weather conditions during the period from 2000 to 0600 hours that preceded every bug collection day were suitable for blood-feeding [20] and mostly so for flight dispersal (≥22°C and wind speed <5 km/h [27].

All (peri)domestic sites positive for *T. infestans* among houses inspected from 20 to 27 October 2003 were considered eligible for detailed blood-feeding studies with emphasis on domestic sites. Time constraints for processing the bugs within 8 h of capture dictated that insects from 64 (22.9%) of the candidate sites in 57 house compounds were processed for body measurements, urine color, nutritional status and bloodmeal sources. These 64 sites were all of the sites which satisfied the time constraints, and were not a sample of a larger number of possible sites. In view of host-feeding and fecundity results, we took a supplementary sample (from frozen specimens) including 57 peridomestic sites from 29 other house compounds to increase the sample size of peridomestic bugs and females to approximately 60 and 30 per ecotope, respectively; this supplementary sample lacked data on urine color, engorgement status and W∶L.

All triatomine bugs collected were kept in a cooler at 10–12°C until arrival to the field laboratory, and then were identified to species and counted by stage as described [14]. Sex identification in fifth-instar nymphs was based on the presence of an immature female reproductive system on the eighth tergite [30], confirmed via morphological differences in the eighth and ninth tergites [31]. Late-stage bugs were defined as fourth instars, fifth instars, adult females, and adult males. All late-stage bugs were weighed individually in an electronic balance (precision, 0.1 mg, Ohaus, Pine Brooks, NJ) and measured from clypeus to abdominal tip with a hand-held vernier caliber accurate to 0.02 mm. Late stages were individually examined for the presence of colorless urine within 8 h of capture using the method developed by Catalá [23]. The site-specific proportion of *T. infestans* that fed during the preceding night (i.e., daily blood-feeding rate) was estimated as a weighted average of the observed proportion of fourth- and fifth-instar nymphs with colorless urine multiplied by a temperature-dependent correction factor and the (uncorrected) proportion of adult bugs with colorless urine, relative to the number of bugs examined for urine color [23]. The underlying physiological rationale of the method, comparison with other data, estimation details and the exact formula are given elsewhere [20].

The engorgement status (formerly called qualitative nutritional status in [10], [32]) of late-stage *T. infestans* was determined by direct observation of a cross-sectional view of the abdomen perpendicular to the long axis of the body to assess the degree of cuticle distension (nymphs) [10] and the volume and shape of the anterior midgut against a flashlight (adults) [33]. Bugs were classified as unfed, little fed, medium fed, and fully fed (i.e., starved, or with scarce, good, or large blood contents). All bugs were kept frozen at −20°C upon arrival to the laboratory in Buenos Aires.

### Host-feeding sources

Bugs were dissected and the midgut with the blood meal was extracted into a previously labeled, weighed vial [20], [32]. The number of chorionated eggs present in the oviducts was counted. Bloodmeal contents were tested with a direct ELISA assay against human, dog, cat, chicken, pig, goat and murid rodent (rat or mouse) antisera with high sensitivity and specificity values as described [20], [32]. We report the proportion of reactive bugs (i.e., those positive against any of the tested antisera) that contained each type of host blood.

### Data management and analysis

The data for each house visited were entered in two databases: one for site infestation (Table S3, data sheet Infestation_data) that included a unique identifier code for each of the 1,297 collection sites at 233 inhabited houses, and one for each bug examined for urine color, body weight (W, mg), total body length (L, mm), W∶L ratio (mg/mm) and other attributes in a sample of sites (Table S3, data sheet Feeding_fecund_flight_data). The latter database included a total of 769 late-stage insects examined for at least one of these attributes: 544 were examined for transparent urine; 551 for W; 550 for L and engorgement status; 729 for ELISA; 214 fifth instars for sex identification, and 216 females for fecundity. All proportions herein reported have attached standard errors clustered by bug collection site as estimated by Stata 12 [34].

The relative abundance of fifth-instar nymphs (the stage with submaximal reproductive value) was taken an an index of successful development over several months and future recruitment of adult stages (i.e., productivity) in infested sites. Total bug catch per infested site, productivity, and female fecundity were highly overdispersed and no transformation normalized the data. Therefore we used negative binomial regression with robust standard errors to test for ecotope effects on the response variables using Stata 12 [34], [35]; relative abundance (RA), relative productivity (RP) and relative fecundity (RF) (labeled in Stata output as ‘incidence-rate ratios’) and their 95% CI were calculated. Logistic regression with robust standard errors was used to test for ecotope effects on site-specific infestation prevalence.

Daily blood-feeding rate and engorgement status (with unfed and little-fed bugs pooled in one class, and medium- or fully-fed bugs in another) were used as response variables in random-intercept logistic regression models clustered by collection site using Stata 12 [34], [35]. Different bug collection sites were assumed independent (i.e., having a domestic site within the same house compound would not affect the blood-feeding rate (or any other demographic parameter) of bugs in the chicken coop of the same compound). The predictor variables were ecotope (a categorical variable with seven levels), bug stage (a categorical variable with three levels, with fourth- and fifth-instar nymphs pooled, males and females), total bug abundance per unit of catch effort per site, and mean maximum temperature during the night preceding bug catch. Interaction terms were added one by one to the main-effects model and retained in the final model if the coefficients of the interaction terms had P<0.05 for a test of the null hypothesis that the coefficient was zero.

We used the allometric equation in log-transformed form (log W = log(a)+b*log L) to estimate the parameters a and b for males, females, fourth- and fifth-instar nymphs using random-intercept linear regression analysis [35]. Throughout log = log_e_. Fourth- and fifth-instar log W distributions were bimodal in every ecotope and could be separated visually into an upper (heavier) and lower (lighter) distribution with separation points at 4.1 and 5.2 mg, respectively. The distributions of log L in fourth- and fifth-instar nymphs were also bimodal and could be separated visually into an upper and lower distribution at 2.55 and 2.85 mm, respectively. We ran separate regressions for the lower and upper distributions for fourth- and fifth-instar nymphs, using the log W separation points for each stage to break each stage's distribution into two parts. In a second step we added ecotope and its interaction with log L as independent variables.

Individual adult W∶L and the maximum temperature between 2000 and 0600 hours of the night preceding capture were used to estimate an individual probability of flight initiation of adult bugs from a given site using the model described in [17]. In practice, such maxima were always at 2000 hours. At this time in October in the same region, the differences between internal and external temperatures within various (peri)domestic ecotopes were nil [9]; therefore we did not adjust records of external temperature for ecotope-specific dampening effects. Adult *T. infestans* bugs with a probability of flight initiation greater than 0.05 were taken as potential fliers. Mark-recapture experiments with adult *T. infestans* conducted in a salt flat in Cordoba recorded that a small fraction of the bugs that flew did so for just a few meters from the release point [26]. Therefore, we assumed that a small fraction (0.05) of the adult bug population that might fly would travel such short distances that they would not count as dispersers. We also tried trivial flight thresholds of 0.01, 0.05, and 0.10 to see how sensitive the results were to the choice of the threshold.

### Quantitative summary of risk indices

#### (a) The number of bugs that fed on humans

For ecotope i (for example, i = 1 refers to domiciles, i = 2 refers to storerooms), let SI(i) be the number of sites infested with *T. infestans*. Let B(i) be the average number of bugs per infested site of ecotope i. Then the total number of bugs in all sites of ecotope i is SI(i)*B(i) = TB(i). Let PR(i) be the percentage of bugs that were reactive for ELISA (i.e., had an identifiable blood meal). Let PH(i) be the fraction of reactive bugs in ecotope i that fed on humans. Then the number of bugs of ecotope i that were reactive and were fed on humans is BH(i) = TB(i)*PR(i)*PH(i) = SI(i)*B(i)*PR(i)*PH(i).

#### (b) The egg production of female bugs

Let AF(i) be the fraction of bugs in ecotope i that are adult females (that is, the fraction of bugs in ecotope i that are adults, times the fraction of adult bugs in ecotope i that are females). Let E(i) be the average number of chorionated eggs per adult female. Then the total eggs in ecotope i is TE(i) = TB(i)*AF(i)*E(i) = SI(i)*B(i)*AF(i)*E(i). This quantity TE(i) estimates the contribution of ecotope i to bug population growth, assuming that each egg contributes equally to bug population growth. Data are lacking on the egg production of bugs in granaries.

#### (c) The number of flight-dispersing females

Let D(i) be the probability that an adult female in ecotope i will disperse by flight in some specified unit of time. Then the total number of flight-dispersing adult females expected per unit of time from ecotope i is TD(i) = TB(i)*AF(i)*D(i). This quantity TD(i) is an estimate of the contribution of ecotope i to the recolonization or reinfestation of uninfested sites by adult female bugs, assuming that each dispersing adult female bug has an equal chance of colonizing an uninfested site. Distance between sites may be relevant when houses are much more sparsely distributed than in the rural villages of Santiago del Estero, where there is a dense network of potential bug habitats. One could define and estimate similar estimates for adult male bugs or for all adult bugs or for all bugs. For goat corrals, for which we had no W∶L data, we borrowed spring estimates from a previous study in the same region [10]. Lack of W∶L data for granaries did not allow us to estimate flight dispersal probabilities from this ecotope.

To calculate risk indices per site (i.e., structure) for each ecotope, we divided the total index by the number of sites of that ecotope (Table S2).

## Results

### Population abundance and stage structure

Of 1,297 identified sites searched for triatomine bugs, 279 (21.5%) were positive for *T. infestans* and 2,145 bugs were caught (Table 1). The ecotopes most frequently infested were domiciles (38.7%), granaries (33.3%), chicken coops (30.2%), storerooms (29.5%), goat corrals (26.6%) and pig corrals (23.9%). In absolute numbers, however, domiciles led the ranking (94) followed by storerooms, goat and pig corrals (41–45), and chicken coops (26). These five ecotopes plus kitchens and granaries (the seven main ecotopes) included 274 of the 279 infested sites detected. Using logistic regression analysis, we rejected the null hypothesis of no differences among main ecotopes in infestation prevalence (Wald χ^2^ = 27.80; df = 6; P<0.001). Domiciles had greater odds of being infested than goat corrals –the reference category (OR = 1.74, CI = 1.13–2.67)– and kitchens were less likely to be infested (OR = 0.43, CI = 0.23–0.79).

**Table 1:** Mean prevalence of site infestation with *T. infestans*, daily host-feeding rate and median feeding interval by ecotope type, and total risk indices (a–c).

Ecotope	Number of sites examined (SE)	Number of sites infested (SI)	% infested PI (SI/SE)	Daily feeding rate (%)	Median feeding interval (in days, quartiles)	Mean number of bugs per infested site (B)	Total bugs (TB)	% of bugs that were reactive (PR)	% of reactive bugs fed on humans (PH)	(a) Number of bugs feeding on humans (BH)	% bugs in ecotope i that are adult females (AF)	Mean number of eggs per adult female (E)	(b) Total eggs of adult females (TE)	Probability that adult female will disperse (D)	(c) Total number of dispersing adult females (TD)
Domicile	243	94	38.7	30.1	4.1 (2.4, 7)	5.78	543	94.8	68.2	351	21.2	12.0	1380	0.064	7
Storeroom	139	41	29.5	26.0	3.1 (2.9,6.3)	8.88	364	95.7	2.2	8	12.8	8.6	430	0.000	0
Kitchen	127	17	13.4	26.7	10.2 (2.4,18.0)	12.82	218	94.4	0.0	0	18.4	12.4	496	0.250	10
Chicken coop	86	26	30.2	31.4	2.8 (2.2,>30)	9.54	248	98.9	0.0	0	9.8	14.8	361	0.000	0
Pig corral	180	43	23.9	33.3	4.0 (1.9, 10.0)	6.02	259	94.1	0.0	0	15.1	10.3	402	0.000	0
Goat corral	169	45	26.6			5.13	231	97.4	2.7	6	35.5	7.7	262	0.000	0
Granary	24	8	33.3			17.00	136	80.0	0.0	0	14.0	0.0	0		
Latrine	98	3	3.1				13								
Open shed	29	1	3.4				131								
Horse corral	4	1	25.0				2								
Other^a^	198	0	0.0				0								
Total	1297	279	21.5	29.6	4.0 (2.4, 7)	7.69	2145								

Figueroa, October 2003 (spring).

a Including 48 mud ovens, 60 trees with or without chickens, 21 piled materials, 48 cow corrals, 13 nests and 8 miscellaneous structures inspected.

CI, confidence interval.

Blanks indicate no data.

**Figure 1:**
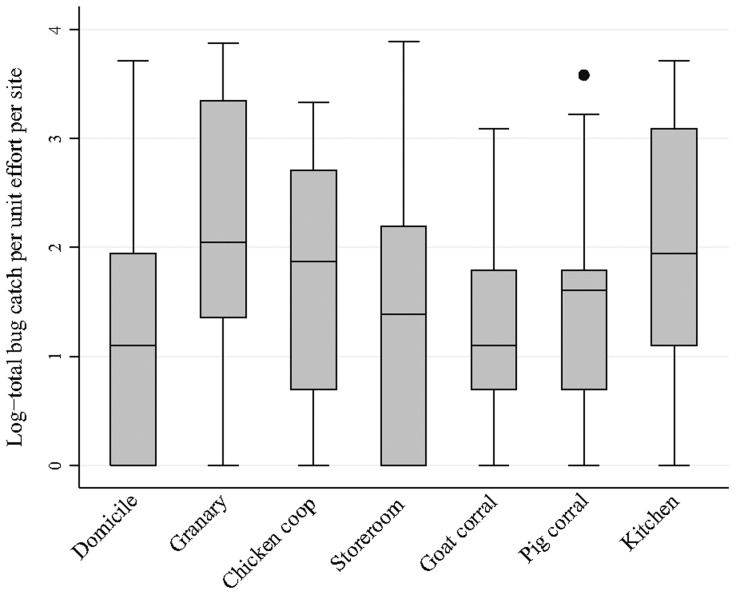
Relative abundance of *T. infestans* per infested site in the 274 infested sites of the seven main ecotopes. Figueroa, October 2003 (spring). In the box-and-whiskers plot, the shaded rectangle spreads from the first to the third quartile, whiskers include up to 1.5 times the interquartile range, and outer dots represent outlying values. Here log = log_e_.

Bug abundance was highly overdispersed in every main ecotope (range of variance-to-mean ratios, 5–15) (Figure 1). Using negative binomial regression analysis, the relative abundance of *T. infestans* per infested site differed significantly among the seven main ecotopes (Wald χ^2^ = 23.58; df = 6; P<0.001). Compared to infested goat corrals, infested granaries (RA = 3.31, CI = 1.52–7.22), kitchens (RA = 2.50, CI = 1.42–4.38), chicken coops (RA = 1.86, CI = 1.18–2.92) and storerooms (RA = 1.73, CI = 1.04–2.87) had significantly larger relative bug abundance. Similarly, in infested sites, productivity was significantly larger in granaries (RP = 4.84, 1.74–13.43), chicken coops (RP = 3.07, 1.71–5.51), kitchens (RP = 2.93, 1.42–6.01) and storerooms (RP = 2.16, 1.17–3.97) than in goat corrals (Wald χ^2^ = 32.0; 6 df; P<0.0001).

**Figure 2:**
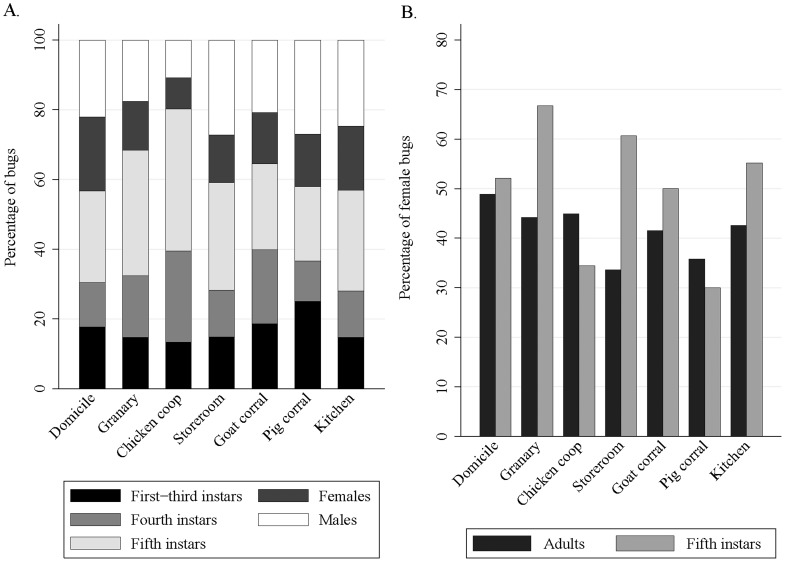
Stage structure of *T. infestans* populations (A) and proportions of adult bugs and fifth-instar nymphs that were female (B) in the 274 infested sites of the seven main ecotopes. Figueroa, October 2003 (spring). Domicile = human sleeping quarters.

The stage structure of *T. infestans* populations (after pooling first to third instars) differed significantly among the seven main ecotopes (χ^2^ = 92.0; df = 24; P<0.001) (Figure 2A). Fifth-instar nymphs comprised the largest fraction of the population in chicken coops (38.8%), granaries (36.0%) and storerooms (30.8%) –all ecotopes where chickens were the main or only bloodmeal source– compared with goat corrals (24.7%) or pig corrals (21.2%). The mean percentage of adult females (41.7%) differed marginally significantly among the main ecotopes (χ^2^ = 11.1; df = 6; P = 0.085), with more females in domiciles (48.9%) and chicken coops (46.2%) than in storerooms (33.6%), pig corrals (35.8%) and goat corrals (41.5%) (Figure 2B). However, the sex ratio of fifth-instar nymphs (n = 214) was 50% and did not differ significantly between ecotopes (χ^2^ = 4.84; df = 4; P = 0.30, excluding granaries and pig corrals) (Figure 2B). Recent apparent colonization attempts were more likely in domestic than peridomestic sites (Text S1).

### Blood-feeding rates

Daily blood-feeding rates averaged 29.6% (Table 1). Median feeding interval was 4.0 d across ecotopes and varied widely from 2.8 d in chicken coops to 10.2 d in kitchens; it was the least variable in domiciles. These estimates are derived from data in this paper, except the estimate for domiciles from [20]. Blood-feeding rates were not significantly associated with ecotope and bug stage (Figure S2), bug abundance per site and mean maximum temperatures during the night preceding bug capture using random-intercept logistic multiple regression analysis (Wald χ^2^ = 8.9; df = 6; 64 sites with 544 observations; P = 0.354).

### Host-feeding indices

Of 729 *T. infestans* tested, 695 (95.1%) had at least one host identification and were considered reactive (Table 2). Of 107 bugs classified as unfed 13 (12.2%) were non-reactive, whereas of 416 bugs classified as fed 7 (1.7%) were non-reactive (Fisher's exact test, P<0.0001, rejecting the null hypothesis that unfed and fed bugs had the same proportion of non-reactive bugs).

**Table 2:** Host-feeding patterns of *T. infestans* according to type of ecotope.

	No. of bugs reactive	Blood source (No., %)							All sources	% unmixed
Ecotope	No. non-reactive	Humans	Dogs	Chickens	Cats	Goats	Pigs	Rodent		
Domicile	289	197	26	63	3	6	4	1	300	96.5
	16	68.2	9.0	21.8	1.0	2.1	1.4	0.3		
Granary	8	0	0	2	3	0	2	1	8	100
	2	0.0	0.0	25.0	37.5	0.0	25.0	12.5		
Chicken coop	86	0	0	86	0	1	0	0	87	98.8
	1	0.0	0.0	100.0	0.0	1.2	0.0	0.0		
Storeroom	89	2	4	73	1	13	0	0	93	96.6
	4	2.2	4.5	82.0	1.1	14.6	0.0	0.0		
Goat corral	74	2	1	6	2	55	16	0	82	90.5
	2	2.7	1.4	8.1	2.7	74.3	21.6	0.0		
Pig corral	64	0	0	0	5	8	52	0	65	98.4
	4	0.0	0.0	0.0	7.8	12.5	81.3	0.0		
Kitchen	85	0	2	82	0	0	2	0	86	98.8
	5	0.0	2.4	96.5	0.0	0.0	2.4	0.0		
Overall	695	201	33	312	14	83	76	2	721	96.7
	34	28.9	4.7	44.9	2.0	11.9	10.9	0.3		

Figueroa, October 2003 (spring).

The reactive bugs fed mainly on chickens (44.9%) and humans (28.9%), and much less frequently on goats (11.9%), pigs (10.9%), dogs (4.7%), and cats (2.0%) (Table 2). Only two rodent bloodmeals were detected and they occurred in a domicile and a granary. The host-feeding patterns were spatially structured according to habitat type and correlated closely with the main resident host(s). In domiciles, the main bloodmeal sources were humans (68.2%) followed by chickens (21.8%) and dogs (9.0%) [20]. Chicken blood meals prevailed in chicken coops, storerooms and kitchens (range, 82.0–100%), and occurred in all ecotopes except pig corrals. Bugs from pig corrals and from goat corrals were mainly fed on pigs and goats, respectively. Domiciles, storerooms and goat corrals had the largest number of different bloodmeal sources identified, and mixed blood meals were most frequent in storerooms (9.5%). Most bugs with identified sources had unmixed blood meals (96.7%) and very few had fed on two (2.9%) or on three (0.4%) host species. No significant association between the percentage of bugs that fed on a given host and bug stage was detected by separate χ^2^ tests.

The occurrence of blood meals from hosts that characteristically did not use certain ecotopes provided clues to bug dispersal events between domestic and peridomestic ecotopes (Text S1).

### Engorgement and nutritional status

**Figure 3:**
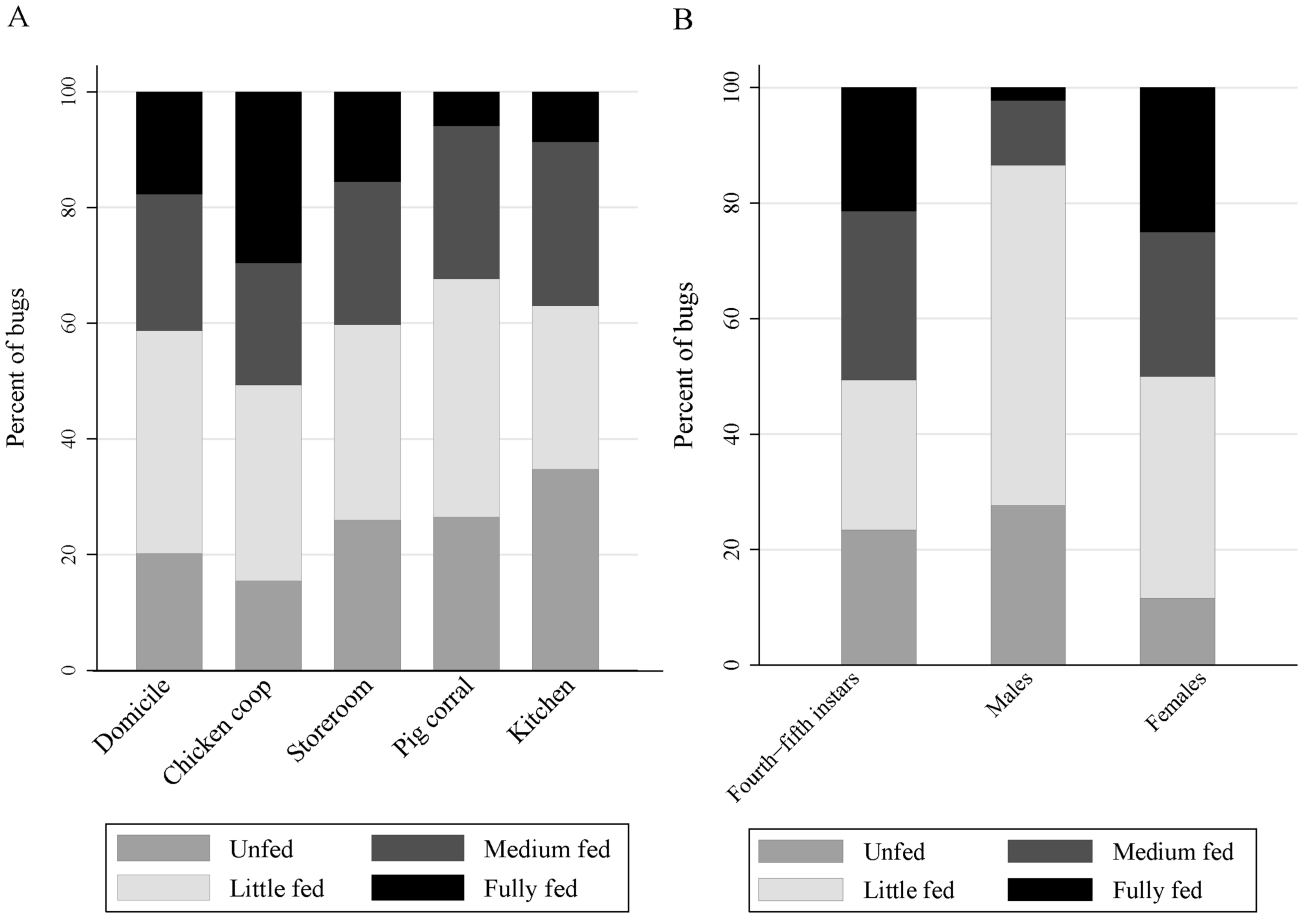
Engorgement status of *T. infestans* by type of ecotope (A) and bug stage (B). Figueroa, October 2003 (spring).

Fully-fed *T. infestans* declined from 29.6% in chicken coops and 17.7% in domiciles to 8.7% in kitchens and 5.9% in pig corrals, with marginally significant differences among ecotopes (χ^2^ = 19.3; df = 12; P = 0.081) (Figure 3A). Conversely, unfed bugs peaked in kitchens (34.8%) and pig corrals (26.5%); they were the fewest in domestic sites (20.2%) and chicken coops (15.5%). Females were more frequently fully or medium engorged than males (Figure 3B). Random-intercept logistic regression analysis of engorgement status showed significant interaction effects (P<0.05) between ecotope and bug stage (Wald χ^2^ = 51.9; df = 14; P<0.001), with female bugs having a six times greater odds of being medium or fully engorged than nymphs (reference category). Engorgement status and W∶L ratios were positively and highly significantly (P<0.001) related among fourth instars (Spearman's correlation coefficient, ρ_S_ = 0.779), fifth instars (ρ_S_ = 0.821), females (ρ_S_ = 0.660), and males (ρ_S_ = 0.526). The proportion of bugs that fed on the preceding night increased steadily from 4.2% in unfed bugs to 20.7%, 38.0% and 68.8% in successive categories of increasing engorgement (χ^2^ = 119.6; df = 3; P<0.001).

**Figure 4:**
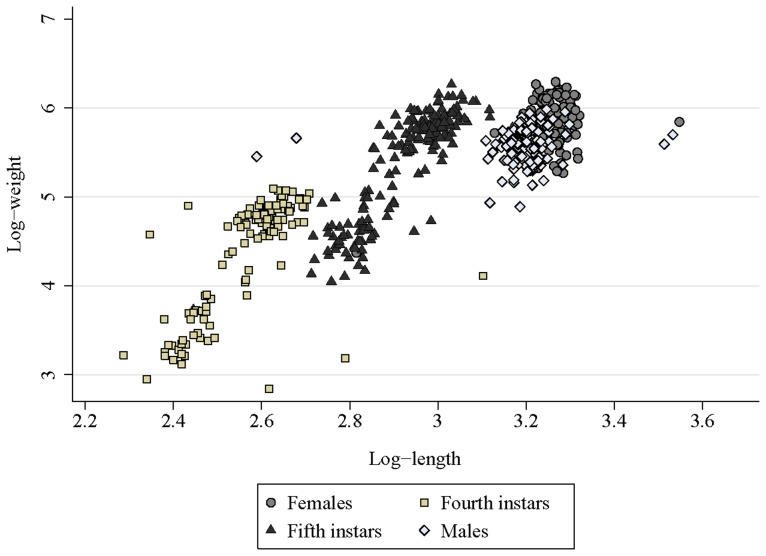
Relationship between log-weight (W) and log-length (L) in different stages of *T. infestans* collected in (peri)domestic ecotopes. Figueroa, October 2003 (spring). Here log = log_e_. Four outlier values for males (with log-length <2.7 and >3.5 mm) and three for females (with log-length <3.2 and >3.5 mm) were excluded from the graph and regression model.

We investigated the relationship between log W and log L and whether it varied with bug stage and ecotope using random-effects multiple linear regression (Table S1). Log W increased highly significantly with log L among the lower and upper distributions of fourth and fifth instars and in females, but not in males (Figure 4). When ecotope effects were added to these models, significant interaction effects (P<0.05) were detected for lighter fourth instars (with greater increase in log W per unit of log L in domiciles relative to kitchens –the reference category); lighter fifth instars (with lower increase in pig corrals), and heavier fifth instars (with lower increase in storerooms).

### Female fecundity

Most females (81%) were gravid (Table 3). Females with no eggs occurred much more frequently in pig corrals and goat corrals (31–32%) than in chicken coops and kitchens (<8%). The fraction of gravid females differed significantly among ecotopes (χ^2^ = 11.8; df = 5; P = 0.038, excluding granaries). The frequency distribution of chorionated eggs per female (including females who were not gravid) was substantially and significantly overdispersed overall and at every ecotope. The mean number of chorionated eggs per female increased from 7.7–8.6 in goat corrals and storerooms to 14.8 in chicken coops (Table 3). Bugs from goat corrals (RF = 0.62, CI, 0.40–0.97) and storerooms (RF = 0.69, CI, 0.51–0.94) had a significantly lower fecundity than those from kitchens (reference category) using negative binomial regression clustered by site (Wald χ^2^ = 14.9; df = 5; P<0.011; n = 214 females). The number of chorionated eggs per female correlated positively and strongly (r = 0.516; P<0.001) with W∶L (Figure S3).

**Table 3:** Frequency of female *T. infestans* with chorionated eggs and number of eggs per female (including females with no eggs), by ecotope.

	No. of females		No. of eggs per female		Variance-to-mean ratio
Ecotope	examined	% with eggs	Mean	95% CI	
Domicile	71	85	12.0	10.1–13.9	5.3
Storerooms	30	83	8.6	6.1–11.0	5.0
Kitchens	28	93	12.4	8.8–16.0	6.9
Chicken coop	25	92	14.8	11.4–18.3	4.7
Pig corral	31	68	10.3	6.8–13.8	9.0
Goat corral	29	69	7.7	3.2–12.2	18.0
Granary	2	0	0.0		
Total	216	81	11.0	9.7–12.2	7.3

Figueroa, October 2003 (spring). CI, confidence interval.

### Flight dispersal

**Figure 5:**
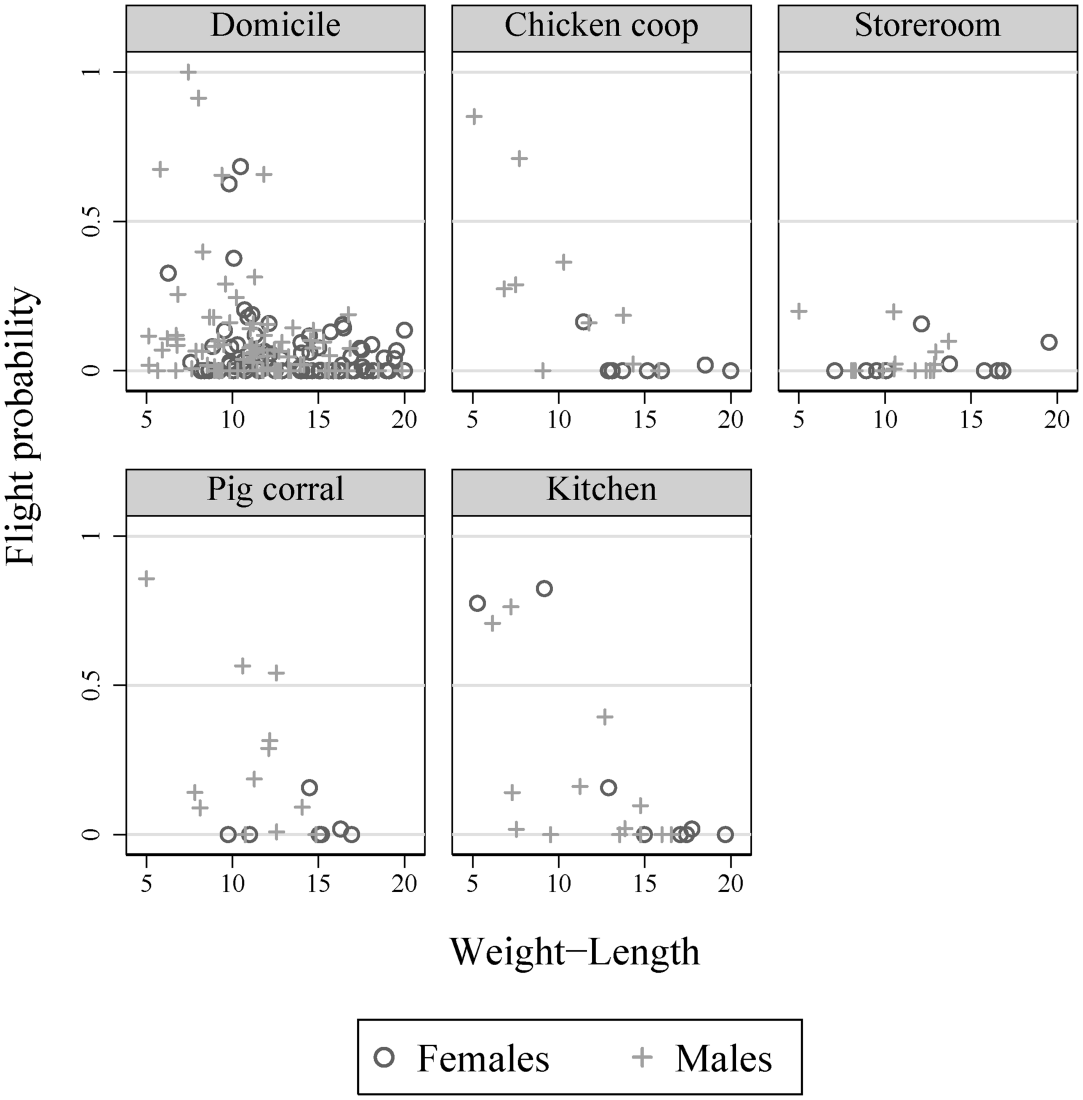
Predicted probability of flight initiation of *T. infestans* by W∶L ratio for adult female (o) and male (+) bugs in domestic sites, chicken coops, storerooms, pig corrals, and kitchens. Figueroa, October 2003 (spring).

The percentage of all female adults that were potential fliers predicted by the model [17] peaked in kitchens (25.0%) and domestic sites (6.4%) and was very low in storerooms, chicken coops and pig corrals in early spring (Figure 5). Potential male fliers outnumbered females at every ecotope and declined from 60.0% in chicken coops and 58.3% in pig corrals to 30.8% in kitchens, 25.0% in domiciles, and 20% in storerooms. Ecotopes ranked differently by sex. The use of a trivial flight threshold of 0.01 (instead of 0.05) yielded a similar qualitative ranking of ecotopes favorable for flight among females (25.0%, 6.4%, 0%, 0% and 0%, respectively) and males (50.0%, 58.3%, 30.8%, 16.7%, and 6.7%, respectively). Nor did higher thresholds (0.1 and 0.15) modify the patterns observed with a threshold of 0.05.

Using the W∶L ranges of observed fliers (females, 5.9–11.1 mg/mm; males, 3.6–10.3 mg/mm) in light-trapping surveys of *T. infestans* [27], we estimated that 17.0% (19 of 112) of all females and 38.8% (52 of 134) of all males measured for W∶L qualified as potential fliers under adequate weather conditions.

### Quantitative summary of risk indices

Table 1 column (a) shows that domiciles had, in total, 351 bugs that were reactive and fed on humans. Storerooms had 8 reactive, human-fed bugs and goat corrals 6. None of the other ecotopes had any reactive, human-fed bugs. The per-site risk index also shows that the overwhelming majority of human-vector feeding contacts occurred in domiciles (Table S2).

Table 1 column (b) shows that the six principal ecotopes other than granaries all produced substantial numbers of eggs. Domiciles had almost three times as many eggs as the next-ranked ecotope, kitchens, followed by storerooms and pig corrals. Goat corrals, though ranked last among the six ecotopes, had about half as many eggs as chicken coops. All six of these ecotopes contributed substantially to bug population growth. Per site, however, chicken coops contributed nearly 50% more eggs than domiciles and 2–4 times more than any other ecotope (Table S2, column b).

Table 1 column (c) shows that the total number of flight-dispersing adult females (per unit effort) expected from kitchens was 10 and from domiciles 7, with no contributions from other main ecotopes. However, flight-dispersing females per kitchen site were expected to be 2.6 times more frequent than flight-dispersing females per domicile site (Table S2).

## Discussion

Our study shows that (peri)domestic populations of *T. infestans* occupying distinct habitats differed substantially and consistently in various fitness-related measures, with chicken coops and domiciles leading the ranking of key habitats. In addition to the characteristic aggregation of bug abundance, we show that female fecundity was also aggregated across ecotopes. Both metrics indicate that bug population growth and abundance were concentrated in a small fraction of very productive, high-quality sites. Our observations pertain to early spring, when bugs achieve maximum rates of population growth [24], temperatures were nearly optimal (reviewed in [9]), and to the third year post-spraying, when the rate of house reinfestation also peaks in the Gran Chaco [4]. Although our data describe a cross-sectional sample for early spring and comparison with other seasons is not possible, the data do challenge the idea that flight dispersal could hardly occur in spring. Both the flight index and host-feeding data support that early spring may represent a pulsed dispersal period of *T. infestans* [29] that our more limited light-trapping experiments did not detect [27]. The flight indices also show that chicken coops, goat and pig corrals were not the only sources of bugs for reinfestation of other structures, with domiciles and kitchens having a prime role in spring. A major strength of the current research effort is the integration of multiple pieces of demographic evidence from all known bug habitats at an unprecedented geographic scale, with special emphasis on habitat suitability and domestic bug populations.

Human sleeping quarters had maximum infestation prevalence rate (38.7%), human-feeding bugs and total egg production, with submaximal values for other demographic and blood-feeding attributes including flight indices. Domestic infestations were high considering the relatively recent insecticide spraying campaign; the fact that professional residual spraying with pyrethroids is known to suppress domestic infestations with *T. infestans* but fails to do so in peridomestic structures [13], [14]; and the local absence of pyrethroid resistance [13], [14]. The much higher apparent recolonization of domiciles relative to peridomestic sites (Text S1) may have been driven by bugs dispersing from peridomestic residual foci attracted to white lights [36], [37]. Then the bugs found suitable refuges in the cracked mud walls and thatched roofs which dampen environmental extremes and have near-optimum average temperatures ranging from 22 to 27°C [9]. These results show again that in the absence of effective vector surveillance and control actions, in this region domestic recolonization progresses rapidly fueled by peridomestic residual foci [4], [11], [13], [38]. The high, least variable blood-feeding rate of domestic bugs was reflected in submaximal engorgement and nutritional status. Because the vector-borne transmission of *T. cruzi* occurs with much more intensity in human sleeping quarters [39], the reduced starvation levels of domestic bugs imply that they are more likely to harbor greater *T. cruzi* densities (including the infectious trypomastigote stage) than other bugs subject to blood-feeding restrictions [40].

Bug populations from chicken coops had several features indicating a high-quality habitat: maximal blood-feeding rates, engorgement status, female fecundity, bug abundance and productivity, and a much lower tendency to disperse by flight than bugs from other habitats in spring, as in previous studies [8], [10], [21]. The higher feeding frequency and engorgement on chickens most likely compensated for the lower nutritional quality of bird versus mammalian blood, with chickens having much lower hematocrit, hemoglobin and plasma protein than mammals [41]. Consistent with this pattern, female *T. infestans* experimentally fed on guinea pigs had higher fecundity and fertility than pigeon-fed bugs [42].

Key regional husbandry practices underlying the observed patterns include that chickens are the most prevalent and abundant domestic host; they are rarely enclosed in a fixed structure; nesting peaks in spring and early summer, and constant nesting locations favor bug feeding on chickens [7], [8], [10]. The transient occupation of chicken nests implies that local bugs may disperse when hosts move away. Bug populations from other ecotopes associated with chickens (granaries, storerooms and kitchens) shared some of the features shown by chicken-coop bug populations though at lower levels, probably because these ecotopes had lower host occupation and density.

Bug populations from pig and goat corrals had lower feeding rates, engorgement status, and W∶L (in lighter fifth instars) than bugs from chicken coops, as before [10], [27]. Here we additionally show that corrals had a much lower proportion of gravid females and female fecundity than bugs from chicken coops, as predicted. Although goat corrals on average had the largest host abundance per site, blood-feeding and nutritional status of bugs were relatively poorer. Pigs are rarely enclosed in corrals, whereas goats may or may not be enclosed at night depending on local husbandry practices and access to the limited grass available in the dry Chaco [10]. In general, the joint action of these factors determines the stability of host supply at the site level, host exposure and host-vector contact rates.

Granaries and chicken coops had greater average bug abundance and productivity than pig or goat corrals, and were therefore of greater relative relevance to Chagas disease. Yet a few sites had disproportionately greater bug abundance than most sites from the same ecotopes, and hence may function as key sources. For example, granaries were frequently infested and had large bug abundance, but they were a very small fraction of all existing sites. The occurrence of granaries depends on local agricultural practices: notoriously absent in a nearby rural area where local peasants did not grow corn [14], [15], they were closely related to human infection with *T. cruzi* elsewhere in the same province [43]. The presence of corn and grain sheds were significantly associated with house or peridomestic infestation with *Triatoma pallidipennis* in Mexico and *R. prolixus* in Colombia [44], [45].Vector control personnel frequently reported to us that householders did not allow granaries to be sprayed with insecticides when loaded with corn, and would not empty them for treatment. In consequence, granaries frequently remained infested after insecticide spraying campaigns, and may contribute disproportionately to vector persistence at certain times of the year. In addition to prevalence of infestation, bug abundance, frequency of occurrence, and likelihood of serving as a source of dispersing bugs (all reflected in the flight indices), the susceptibility and access of a given ecotope to control interventions also determines its relative importance for vector suppression efforts.

The median feeding interval of *T. infestans* was very short (4.0 d) and varied more widely across ecotopes than in studies covering a limited habitat range [8], [10], [19]. Bugs from chicken coops fed substantially more often (every 2.8 d) than bugs from other habitats, as predicted by earlier studies. This high feeding frequency is consistent with experimental work showing that when offered daily access to a live rabbit, *T. infestans* and *R. prolixus* females would feed every three days [46], and *T. infestans* females from experimental chicken huts would feed every 1.9 days [23]. In our study, blood-feeding rates were not significantly associated with any of the factors investigated, including overnight temperatures preceding bug catches, unlike in longer experimental studies with more variable temperatures [23]. Female bugs fed much more often than other stages (Figure S2) and apparently had a larger degree of engorgement in chicken coops, partly because they held many eggs.

The fecundity of female populations of *T. infestans* was submaximal and least variable in domestic sites, where total egg production was maximal, and was strongly and positively related to W∶L. These novel findings are partly connected to the absence of previous estimates of fecundity across a wide diversity of habitats and bug populations from a well-defined area. The only published field study on the W∶L distribution of domestic *T. infestans* did not detect a significant association between W∶L on capture and female fecundity measured over the next 30 days [16], perhaps because the sample size was limited (n = 46 females). In bug populations from chicken coops elsewhere, both the weight and female fecundity of *T. infestans* (n = 78) varied little between seasons until the end-of-summer peak of emergence of virgin females [8], implying a weak or nonsignificant relation between W∶L and female fecundity.

The host-feeding patterns of *T. infestans* were spatially structured according to habitat type and correlated closely with the main resident host(s) in each ecotope, as predicted, yet bug mobility between ecotopes was greater than expected. The human-fed bugs detected in peridomestic structures and the pig- or goat-fed bugs in human sleeping quarters are strong evidence of flight or walking dispersal of late-stage nymphs and adults of *T. infestans* in both directions (Text S1). The alternative hypotheses (i.e., some humans used peridomestic structures as nocturnal resting sites; goats and pigs were stationed transiently in domiciles for protection) were highly unlikely; both practices were neither reported by householders nor ever recorded by us during site searches for bugs. Host-feeding results [47], spatial analysis of reinfestation patterns [15] and W∶L distributions [10], [21], [27] consistently support that pig and goat corrals are important sources of bugs that invade domestic habitats. Because corrals or chicken coops with flimsy structure have little capacity to dampen extreme climatic variations [9], bugs at these sites are exposed to increased risks of hyperthermia and desiccation that may trigger bugs' dispersal in search of a blood meal.

Flight-dispersing triatomine bugs nearly always include unfed insects [17], [26], [27], [48], [49], and in consequence their previous blood meals are rarely observable with current methods. This fact, combined with limited samples of tested *T. infestans* and seasonality in flight dispersal intensity [29], may explain the marginal occurrence of human blood meals (range, 1–6%) in peridomestic bugs and of goat-fed bugs (range, <1–4%) in domiciles [8], [47], [50]–[52]. Mobility of *T. infestans* between (peri)domestic structures was also underestimated by limited mark-recapture experiments conducted when temperatures were not favorable for active dispersal [53]. Conversely, *T. infestans* sustained tethered flights for at least 20 min at speeds of 2 m/s [54], suggesting its flight range may exceed 2,400 m. Light-trap sampling, microsatellite markers and wing geometric morphometry also suggested frequent active dispersal of *T. infestans* among neighboring house compounds in this region [27], [55]–[57].

Walking dispersal of nymphs and adult bugs may also play a substantial role in house reinfestation at a finer scale [27], [49], [58]. Fourth- and fifth-instar nymphs of *T. infestans* that walked 8–42 m toward light traps had W∶L values of 2.51 and 6.6–16.67 mg/mm, respectively [27]. Based on the wide range of W∶L recorded here, we infer that a large fraction of late-stage nymphs may disperse actively under suitable conditions and contribute to the spatial aggregation of infestation recorded within or between neighboring house compounds [15], [29], [58]. Active dispersal of triatomine bugs links the network of patches at a local scale to a much greater extent than has usually been assumed [1], [3], [53].

Imbalanced sex ratios may affect insect population dynamics [59]. Sex-biased flight dispersal may contribute to the imbalanced sex ratios among ecotopes, with female bugs outnumbering males in domestic sites and chicken coops. Males outnumbered females in storerooms and pig or goat corrals, as in other surveys [8], [10], [27]. In contrast, females of *Triatoma dimidiata* predominated across (peri)domestic and sylvatic habitats throughout most of the year [22]. In general, variations in standing adult sex ratios depend on the sex ratio at birth, sex-specific survivorship schedules and timing of adult emergence, sex-biased adult dispersal, and the timing of such dispersal [59]. Our results rule out differential sex ratios in fifth-instar nymphs as a contributing cause of imbalanced adult sex ratios. Because males tend to emerge later and survive better than female *T. infestans* in the insectary, the adult sex ratio is expected to be skewed toward males. We estimate the stationary sex ratio in this species as 38% females (calculated from the ratio between life expectancies of each sex in the insectary [1], multiplied by the sex-ratio at birth, assumed to be 1). This estimate is very close to the 37.5% of live *T. infestans* females recorded after house demolition [60]. In our study, however, there was at least a 10% relative excess of adult females in domestic sites relative to the expected stationary sex ratio, and to the observed adult sex ratio in some peridomestic ecotopes.

The hypothesis of female-biased adult dispersal is supported by field observations of individually marked adult *T. infestans* in open chicken coops with many chickens [61] and microsatellite-based genetic studies [22], [55], [57]. Conversely, timed manual catches with a tetramethrin-based dislodgant spray were not male-biased [62]. Therefore, the observed deviations from stationary adult sex ratios may be tentatively attributed to: 1) increased survival of females in domiciles; 2) female-biased out-migration from suboptimal peridomestic habitats, or 3) both. Carefully designed experiments are needed to distinguish between these hypotheses, and to clarify whether reproductive and engorgement status may modify the response of adult bugs to the dislodging spray.

Another novel, unexpected study finding is that the predicted number of potential female fliers per unit of capture effort peaked in kitchens and domestic sites relative to goat or pig corrals, thus rejecting our hypothesis. The disagreement may be explained by the fact that previous studies focused on flight probabilities did not include domestic sites and kitchens for comparison; and did not consider ecotope- and sex-specific variations in adult bug abundance to estimate the number of potential fliers.

In support of current predictions, light-trap experiments revealed repeated flight dispersal events of *T. cruzi*-infected *T. infestans* out of human sleeping quarters during late summer, and that male fliers outnumbered female fliers [27]. Bugs from kitchens may be also pushed to disperse by the repellant effects of smoke. Predictions were not affected by assumptions on the trivial flight threshold even though the model underestimates the observed proportions of potential fliers and does not consider adult sex and wind effects [17], [28]. Although female *T. infestans* from our study area had flight muscles more often than males and more proneness to initiate flight [28], they had greater W∶L and thus were predicted to initiate flight substantially less often than males in spring. Male *R. prolixus* bugs increased substantially their take-off activity in response to female pheromones but the reverse did not occur [63].

Some aspects of our study limit the interpretation of results. To measure female fecundity, we counted the total number of chorionated eggs in the females' reproductive tract rather than recording the rate of oviposition on arrival to the laboratory, based on the assumption that both metrics would be closely correlated. The great majority (91–100%) of *T. infestans* females collected in the dry Argentine Chaco in October–December had sperm in their spermatheca [8], [49], and of the thousands of eggs collected after demolishing a rural house only 5% were non-viable [60]. Even if females had mated, there is a chance that the chorionated eggs would not be fertilized subsequently. How much this may modify the estimates of female fecundity is unclear.

The direct ELISA test is a cost-effective option for processing a sizable number of bugs exposed to a well-known range of domestic host species; its advantages and shortcomings relative to molecular methods were discussed elsewhere [20]. We have not assessed the links between blood-feeding rates, host choices, habitats and vector infection with *T. cruzi*, yet bug infection is restricted to domiciles, kitchens and storerooms [39]. *T. cruzi* is considered to be subpathogenic to triatomine bugs, with infection affecting minimally their vital rates (but not the excretion rates) only under prolonged starvation [40], [64]. Whether infection with *T. cruzi* enhances, inhibits, or does not affect the dispersal of triatomine bugs [65], [66] and specifically of *T. infestans* is crucial for modeling parasite transmission and appears to be unknown.

Host occupancy per site is variable over days to months (especially for chickens, kitchens and storerooms) and difficult to assess precisely [7], [10], [32], which limits our understanding of host-feeding choices and related metrics. The classification of sites according to the local presence or absence of hosts (past and current) or by local host abundance is as important as the classification according to ecotope but presents special challenges in some peridomestic sites (Text S1).

The three summary indices in this analysis (number of bugs fed on humans, total eggs, and total number of dispersing adult females, by ecotope) depend on the mean number of bugs per infested site, B(i), which is assumed to be proportional to the mean absolute bug population size in ecotope i [67]. Clearly B(i) depends on the time and effort devoted to sampling. These summary indices are relative measures useful for comparing ecotopes approximately, but not for comparing studies with different levels or techniques of bug sampling. The total and per-site risk estimates are useful for different purposes; the latter, for example, may be used to compare villages that differ in their relative number of sites per ecotope.

The quantitative comparisons in Table 1 must be treated as approximate, for several reasons. B(i) depends on two assumptions: infested sites of every ecotope have the same probability of being detected as infested, and a bug in an infested site has an equal probability of being counted for all ecotopes, i.e., a bug in an infested kitchen is as likely to be seen as a bug in an infested domicile. The observed infestation indices were most likely underestimated since timed manual bug collections fail to detect very low-density infestations [12], [67], [68]. We used the same search effort in each peridomestic site, 0.25 person-hours, so bug abundance was measured on the same scale for every peridomestic ecotope. Relative to each peridomestic site, we used twice as much search effort, 0.5 person-hours, in each domestic site. Because of differences in size and complexity, we have assumed that estimates of the prevalence of infestation were roughly equivalent between domestic and peridomestic sites (Text S1).

### Implications for vector control and disease transmission

Our study illustrates a demographic approach to identifying key source habitats which may be applied to other seasons, regions, and species of triatomine bugs. For nearly all of them there is a very limited quantity and quality of demographic data that may assist in understanding their population dynamics and response to vector control actions. This information is vital for the design of innovative, improved vector control actions in key ecotopes where bugs achieve maximum fitness. We anticipate that our framework is relevant for the control of *Triatoma brasiliensis* in Brazil, *T. pallidipennis* in Mexico, and *T. dimidiata* in parts of Central America, all of which have extensive peridomestic bug populations that tend to invade human sleeping quarters [69]–[71].

Domestic sites and chicken coops provide higher-quality habitats than other ecotopes, and are therefore involved in the rapid recovery of *T. infestans* populations after control interventions. Human sleeping quarters would serve as prime targets of dispersant bugs after insecticide spraying campaigns that do not achieve community-wide vector elimination, and then would become productive habitats and sources for bug propagation. This dual functioning of human sleeping quarters (as targets and as sources) and its early role in reinfestation has been overlooked in the past.

Domiciles outnumbered each type of peridomestic ecotope in the absolute and relative frequencies of infested sites, but taken collectively peridomestic structures outnumbered infested domestic sites by a factor of three. Various lines of evidence consistently indicate that peridomestic foci bolster vector persistence after insecticide spraying and increase the risk of domestic invasion and recolonization in the Argentinean Chaco [7], [11]–[13], [15], [38]. Some structures such as goat corrals may serve as productive habitats at certain times depending on local host supply, refuge availability and weather, and then become sources when conditions become harsh. We propose that the mosaic of suitable patches in peridomestic areas would shift over seasons, whereas human sleeping quarters provide more stable habitats and near-optimum conditions with a more stable supply of suitable hosts.

This article demonstrates that focusing control efforts on the three ecotopes (domiciles, storerooms, and goat corrals) that housed reactive, human-fed bugs would neither eliminate the substantial contributions to bug population growth from kitchens, chicken coops, and pig corrals nor stop dispersal of adult female bugs from kitchens. Rather, comprehensive control of the linked network of ecotopes in a typical house compound and community is required to prevent feeding on humans, bug population growth, and bug dispersal simultaneously.

For the elimination of *T. infestans* and sustained interruption of parasite transmission, one of the worst things vector control programs can do during the attack phase is to restrict the residual application of insecticides to human sleeping quarters and exclude peridomestic structures; or to replace full-coverage insecticide spraying with selective treatments that have little or no residuality (fumigant canisters, pour-on insecticides) and whose effectiveness at a public health scale has weak or no support, as some of them did to reduce costs and increase apparent coverage albeit with much lower quality and impact [5], [72]. For example, vector control programs that did not spray insecticide on peridomestic structures but used pour-on insecticides on dogs, goats and chickens did not suppress peridomestic infestations [49, p. 233]. There is often a large difference between standard recommendations to vector control programs [73] and the realities of vector control during recent decades, in conjunction with the increasing decentralization of health services and reduced operational capacity in the field. Good practices in vector control need to be further promoted [74]. For elimination purposes in high-risk areas such as the Gran Chaco, improved vector control at present should include full-coverage, professional application of a double dose of suspension concentrate pyrethroid insecticides to main peridomestic ecotopes and single (standard) doses to domiciles and other lower-quality habitats [13], with additional re-treatment of key ecotopes at defined time intervals. For more sustainable vector and disease control, however, improved vector control needs to be combined with long-term investment in improved housing and appropriate animal husbandry [4], [6], [73], [75] in the frame of broad social participation and education.

## Supporting Information

Figure S1 **Map of the study area.** Inset shows locations of Santiago del Estero Province within Argentina. The study communities (white dots) included were: (1) Tres Pozos, (2) Barrio San Francisco, (3) Cardón Esquina, (4) Barrio Nueva Esperanza, (5) Barrio Guadalupe, (6) Barrio Sagrada Familia, (7) La Loma, (8) Invernada Norte, (9) Vaca Huañuna, (10) Bajo Sequeira, (11) El Chañar, Figueroa, October 2003.
(TIF)

Figure S2 **Daily host-feeding rate of** ***T. infestans*** **according to type of ecotope and bug stage.** Figueroa, October 2003 (spring).
(TIF)

Figure S3 **Chorionated eggs per individual female of** ***T. infestans*** **according to her W∶L ratio and ecotope.** Figueroa, October 2003 (spring). Excludes one outlier with W∶L = 24 mg/mm and 62 eggs.
(TIF)

Table S1 **Random-intercept regression equations for the relationship between log_e_-weight (W) and log_e_-length (L) in different stages of** ***T. infestans*** **collected in (peri)domestic ecotopes.** Figueroa, October 2003 (spring).
(DOCX)

Table S2 **Per-site risk indices of the number of bugs that fed on humans, egg production and number of flight-dispersing females.** Figueroa, October 2003 (spring).
(DOCX)

Table S3 **Infestation and stage-specific bug abundance per site; blood-feeding, nutritional and engorgement status and bloodmeal identification results of (peri)domestic** ***T. infestans*** **.** Figueroa, October 2003 (spring).
(XLSX)

Text S1 **Description of ecotopes, host abundance and site occupancy, recent colonizations and evidence of bug mobility between ecotopes.**
(DOC)
