# Supplementary figures and images for: Key Source Habitats and Potential Dispersal of Triatoma infestans Populations in Northwestern Argentina: Implications for Vector Control
Source: PLoS Negl Trop Dis. 2014 Oct 9;8(10):e3238. doi: 10.1371/journal.pntd.0003238 (PMC4191936; doi:10.1371/journal.pntd.0003238)

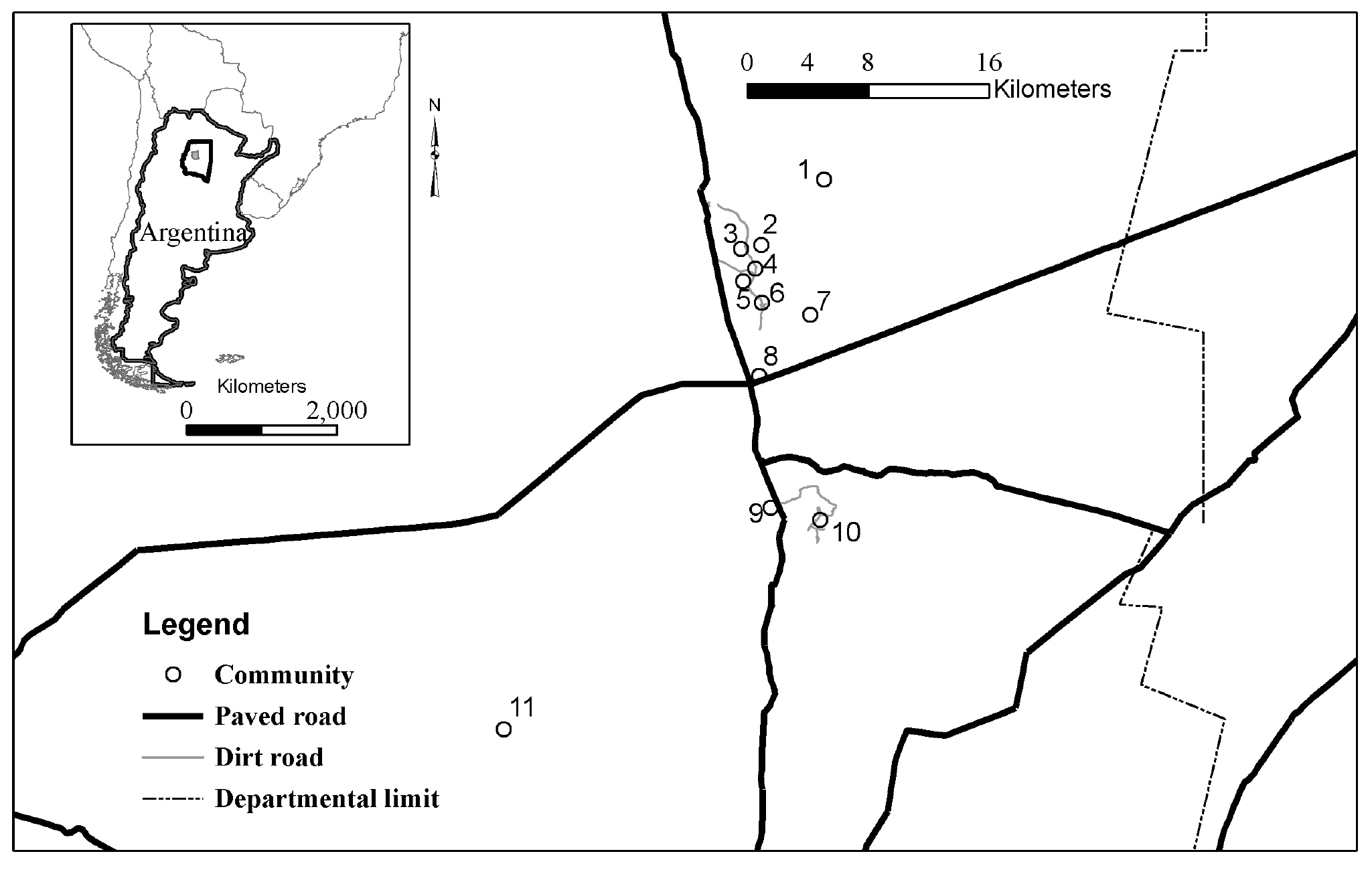

Supplement: Figure S1 — Map of the study area. Inset shows locations of Santiago del Estero Province within Argentina. The study communities (white dots) included were: (1) Tres Pozos, (2) Barrio San Francisco, (3) Cardón Esquina, (4) Barrio Nueva Esperanza, (5) Barrio Guadalupe, (6) Barrio Sagrada Familia, (7) La Loma, (8) Invernada Norte, (9) Vaca Huañuna, (10) Bajo Sequeira, (11) El Chañar, Figueroa, October 2003. (TIF) [file pntd.0003238.s001.tif]

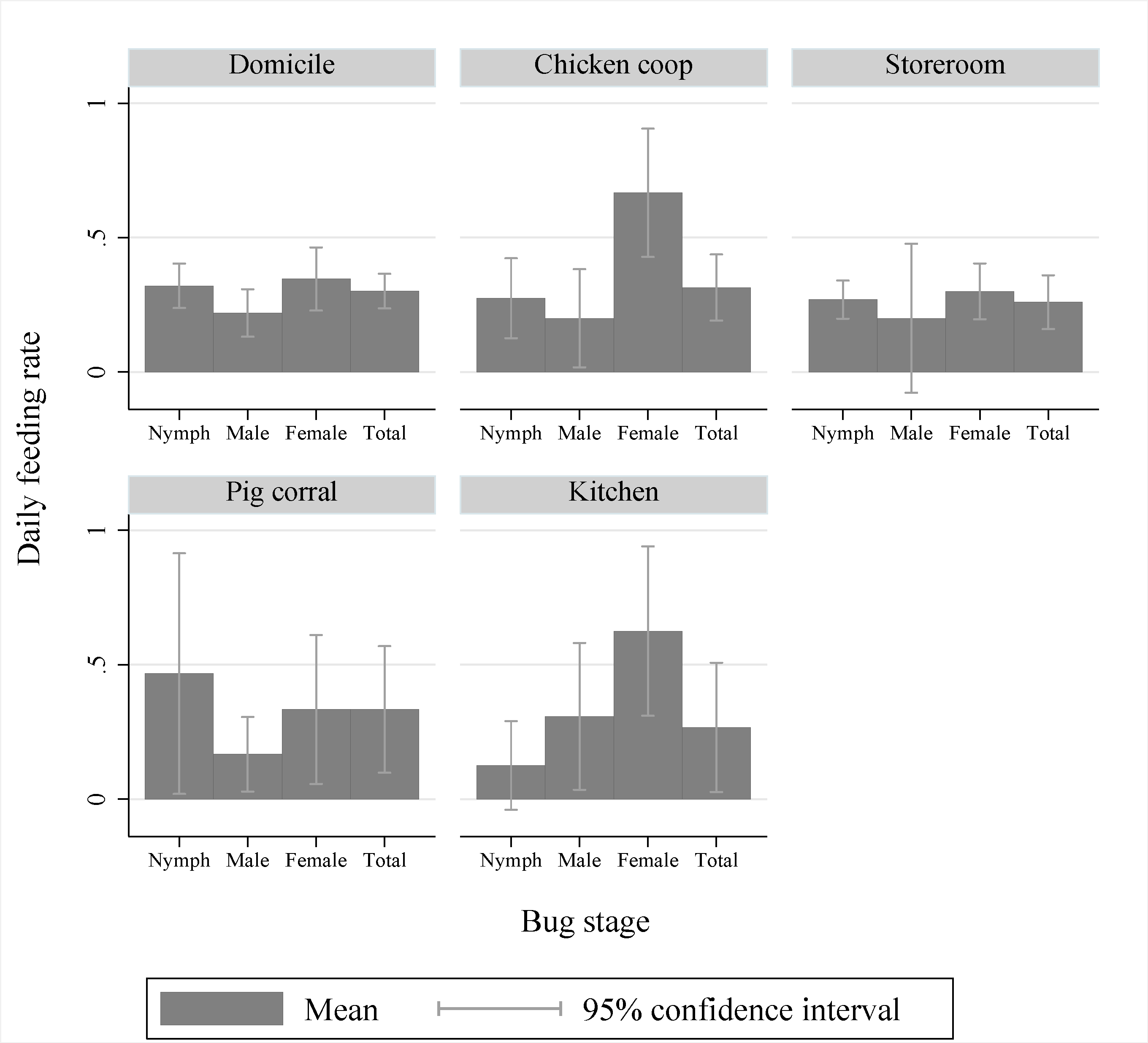

Supplement: Figure S2 — Daily host-feeding rate of T. infestans according to type of ecotope and bug stage. Figueroa, October 2003 (spring). (TIF) [file pntd.0003238.s002.tif]

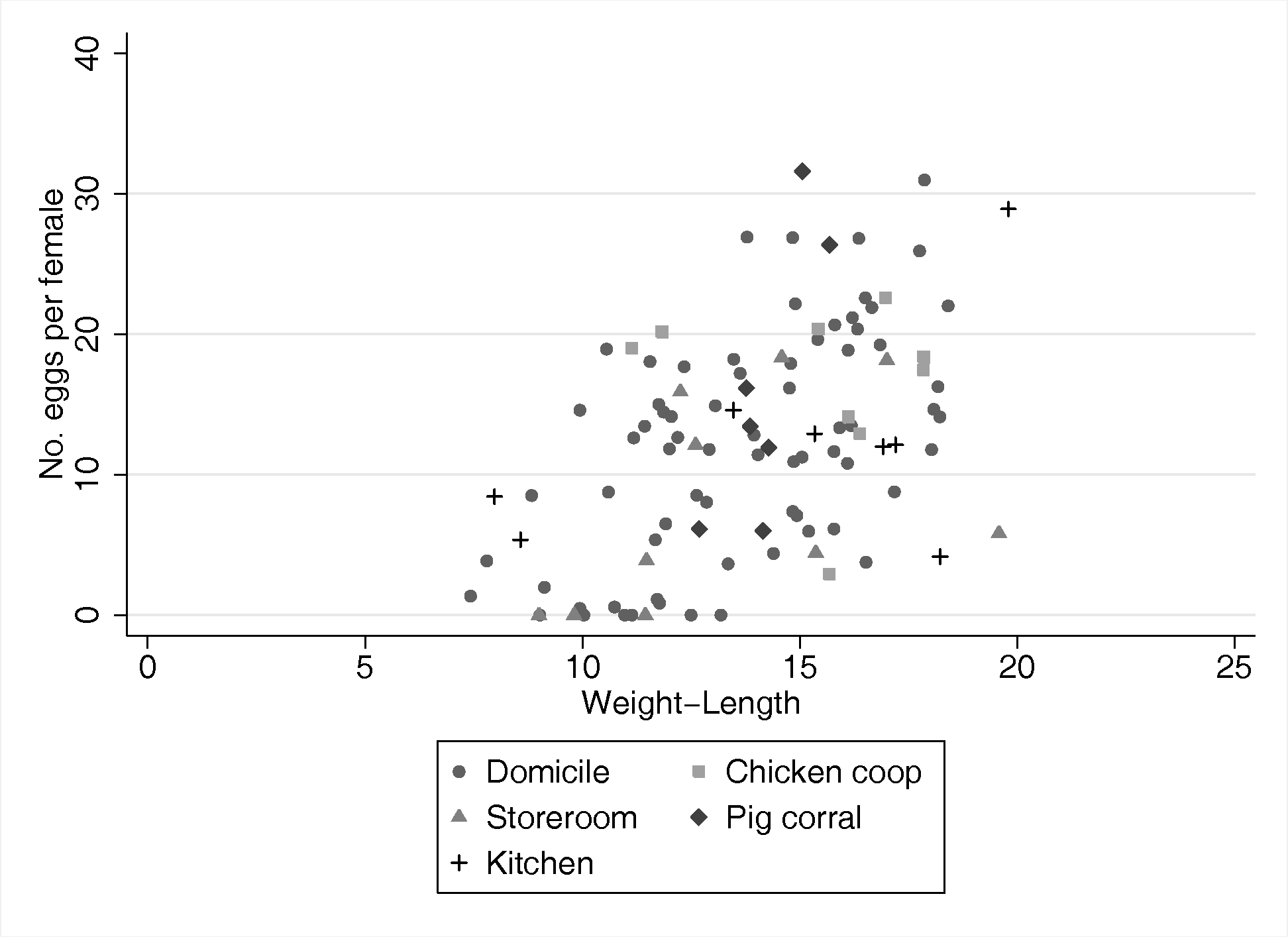

Supplement: Figure S3 — Chorionated eggs per individual female of T. infestans according to her W∶L ratio and ecotope. Figueroa, October 2003 (spring). Excludes one outlier with W∶L = 24 mg/mm and 62 eggs. (TIF) [file pntd.0003238.s003.tif]
