# Supplementary material for: Key Source Habitats and Potential Dispersal of Triatoma infestans Populations in Northwestern Argentina: Implications for Vector Control
Source: PLoS Negl Trop Dis. 2014 Oct 9;8(10):e3238. doi: 10.1371/journal.pntd.0003238 (PMC4191936; doi:10.1371/journal.pntd.0003238)
Supplement: Table S1 — Random-intercept regression equations for the relationship between loge-weight (W) and loge-length (L) in different stages of T. infestans collected in (peri)domestic ecotopes. Figueroa, October 2003 (spring). (DOCX) [file pntd.0003238.s004.docx]

**Table S1** **Random-intercept regression equations for the relationship between log_e_-weight (W) and log_e_-length (L) in different stages of *T. infestans* collected in (peri)domestic ecotopes**. Figueroa, October 2003 (spring).

|  | No. of bugs measured | Regression coefficients | | | Wald χ^2^ test |
| --- | --- | --- | --- | --- | --- |
| Stage | (No. of collection sites) | Intercept (SE) | Slope (SE) | R^2^ | P |
| Male | 130 (43) | -0.5741 (1.5813) | 1.9204 (0.4947) | 0.060 | <0.001 |
| Female | 109 (44) | 2.3472 (2.9750) | 1.0806 (0.9115) | 0.016 | >0.2 |
| V lower | 60 (24) | -2.6741 (1.1442) | 2.5878 (0.4068) | 0.418 | <0.001 |
| V upper | 127 (35) | -0.4728 (0.7378) | 2.0859 (0.2471) | 0.354 | <0.001 |
| IV lower | 37 (17) | 1.1247 (0.7931) | 0.9532 (0.3217) | 0.113 | <0.01 |
| IV upper | 79 (27) | 0.5080 (0.8417) | 1.6228 (0.3220) | 0.277 | <0.001 |

IV upper distribution had weight ≥ 4.2 mg and <5.2 mg.

IV lower distribution had weight <4.2 mg.

V upper distribution had weight ≥5.2 mg.

V lower distribution had weight <5.2 mg.

Females included had length >3.15 cm and <3.5 cm.

Males included had length >3 cm and <3.4 cm.
