# Supplementary material for: Key Source Habitats and Potential Dispersal of Triatoma infestans Populations in Northwestern Argentina: Implications for Vector Control
Source: PLoS Negl Trop Dis. 2014 Oct 9;8(10):e3238. doi: 10.1371/journal.pntd.0003238 (PMC4191936; doi:10.1371/journal.pntd.0003238)
Supplement: Table S2 — Per-site risk indices of the number of bugs that fed on humans, egg production and number of flight-dispersing females. Figueroa, October 2003 (spring). (DOCX) [file pntd.0003238.s005.docx]

**Table S2** **Per-site** **risk indices of the number of bugs that fed on humans, egg production and number of flight-dispersing females.** Figueroa, October 2003 (spring).

| Ecotope | number of sites examined SE | (A) number of bugs feeding on humans BH per site | Relative ratio (A) | (B) eggs of adult females TE per site | Relative ratio (B) | (C) number of dispersing adult females TD per site | Relative ratio (C) |
| --- | --- | --- | --- | --- | --- | --- | --- |
| Domicile | 243 | 1.44 | 40.2 | 13 | 3.0 | 0.07 | 1.0^a^ |
| Storeroom | 140 | 0.06 | 1.7 | 8 | 1.8 | 0.00 |  |
| Kitchen | 127 | 0.00 |  | 9 | 2.1 | 0.18 | 2.6 |
| Chicken coop | 86 | 0.00 |  | 19 | 4.4 | 0.00 |  |
| Pig corral | 180 | 0.00 |  | 5 | 1.2 | 0.00 |  |
| Goat corral | 169 | 0.04 | 1.0^a^ | 4 | 1.0^a^ | 0.00 |  |
| Granary | 24 | 0.00 |  | 0 | 0.0 | ND |  |
|  |  |  |  |  |  |  |  |

^a^ Reference category.
