# Supplementary material for: Key Source Habitats and Potential Dispersal of Triatoma infestans Populations in Northwestern Argentina: Implications for Vector Control
Source: PLoS Negl Trop Dis. 2014 Oct 9;8(10):e3238. doi: 10.1371/journal.pntd.0003238 (PMC4191936; doi:10.1371/journal.pntd.0003238)
Supplement: Text S1 — Description of ecotopes, host abundance and site occupancy, recent colonizations and evidence of bug mobility between ecotopes. (DOC) [file pntd.0003238.s007.doc]

Text S1

Description of ecotopes, host abundance and site occupancy

Storerooms and kitchens (mean area, 12 m^2^) were built similarly to human habitations and were 5-15 m distant from them. Kitchens always had a cooking fire using wood or charcoal (“fogón”). Most chicken coops (0.5 to 25 m^2^ in size, 5 to 50 m from the domicile) had walls of wood sticks or mud bricks and thatched or wood-stick roofs; only a few chicken coops had chicken-wire fencing. Other structures made for sitting chickens (with two or three layers of bricks arranged in a small square or circle) were included in the class ‘chicken coops’. Goat corrals (mean size, 80 m^2^) usually had a fence made with piled thorny shrubs and vertical posts, and a small thatched enclosure for kids. Pig corrals (mean size, 7 m^2^) usually had solid walls and roofs of wooden planks or trunks, sometimes thatched roofs, and usually were the farthest-away peridomestic structures. Granaries ("trojas", mean size, 4 m^2^) were wooden structures used for storage of corn and frequently had sitting chickens associated. Most houses had chickens (99%), dogs (95%), goats (90%), pigs (88%), and fewer had horses or mules (60%), cats (57%) and cows (28%); a median study household had 5 people (first-third quartiles, 4-8), 2 dogs (2-3), 0.5 cat (0-1), 15 chickens (7-22), 11 goats (0-29), 2 pigs (1-6) and 2 horses or mules (0-4); very young chickens were recorded in 71% of houses.

Nearly all of the study houses were permanently inhabited and therefore had the usual domestic animals in their respective structures. The great majority of the bugs were collected from sites used by some bird or mammal host. Although we recorded the occurrence of host species in particular sites (e.g., chickens nesting) on the sketch map or questionnaire during bug searches, domestic animals frequently were wandering around or in the forest during the daytime and assessing current (or past) site occupancy by given host species was rather uncertain. Bugs deprived of hosts (especially late-stage nymphs) may survive for weeks or months in houses or structures left vacant and may disperse. Abandoned houses are sometimes used for shelter by goats and other domestic hosts and the residual bug populations may persist indefinitely.

Recent colonizations

Twenty-one (7.5%) infested sites only had 1-2 male *T. infestans* collected and 23 (8.2%) only 1-2 females. In the context of growing reinfestation after an insecticide spraying campaign, most of these sites likely represented recent colonization attempts. These assumed newly colonized sites constituted 27.7% of the 94 infested domiciles detected and 9.7% of the 186 infested peridomestic sites (Fisher's exact test, P < 0.0001), rejecting the null hypothesis of no difference in the proportion of assumed new colonizations between infested domiciles and infested peridomestic sites. Relative to uninfested sites, the odds of a site being newly colonized was 8.42 (CI, 4.31-16.70) times greater in domiciles (14.9% of 175) than in peridomestic sites (2.0% of 887, Fisher's exact test, P < 0.0001).

Evidence of bug mobility between ecotopes

Four human-fed bugs (2 males, 1 female, 1 fifth instar) were detected in storerooms and goat corrals. Four pig-fed bugs (from each late stage) occurred in domiciles from four different houses that had 1-3 pigs, two of which had an infested pig corral. Six (26%) goat-fed bugs (2 males and 4 fourth or fifth instars) occurred in four domiciles; four of these bugs also had fed on human, dog or chicken. All of these bugs qualified as apparent dispersants. Thirteen late nymphal or adult stages with goat blood meals were detected in a heavily infested storeroom 60 m from an uninfested corral that housed 170 goats; another goat-fed female bug occurred in a chicken coop of a house with >30 goats. All goat-fed bugs occurred in households with goats reported, whereas three pig-fed bugs occurred in two houses having no pigs at the time of the survey.

Comparison of catch efficiency between and within ecotopes

Even different sites within each ecotope are subject to some variation; for example, some houses have smooth, plastered walls, so bugs are in the beds or roofs. It is much easier to catch a bug from a small chicken coop that can be dismantled than from a thatched roof in the interior of a dark place such as a bedroom or storeroom. A human bed is very close to a chicken nest in terms of catch efficiency, and the thick, thorn fences of goat corrals are more difficult places to search for and catch bugs. Bug collectors know of bug preferences for certain spots, and therefore direct their search efforts as a predator, using fecal smears as clues of where bugs are.
